# Evidence of the Anomalous Fluctuating Magnetic State by Pressure-Driven 4f Valence Change in EuNiGe_3_

**DOI:** 10.1021/acs.jpclett.2c03569

**Published:** 2023-01-24

**Authors:** K. Chen, C. Luo, Y. Zhao, F. Baudelet, A. Maurya, A. Thamizhavel, U. K. Rößler, D. Makarov, F. Radu

**Affiliations:** † National Synchrotron Radiation Laboratory, University of Science and Technology of China, Hefei 230026, Anhui, China; ‡ Helmholtz-Zentrum Berlin für Materialien und Energie, Albert-Einstein-Strasse 15, 12489 Berlin, Germany; § Center for High Pressure Science and Technology Advanced Research (HPSTAR), Shanghai 201203, China; ∥ Synchrotron SOLEIL, L’Orme des Merisiers, Saint-Aubin-BP48, 91192 GIF-sur-Yvette, France; ⊥ Department of Condensed Matter Physics and Materials Science, Tata Institute of Fundamental Research, Colaba, Mumbai 400005, India; # Leibniz-Institut für Festkörper- und Werkstoffforschung Dresden e. V. (IFW Dresden), 01069 Dresden, Germany; □ Helmholtz-Zentrum Dresden-Rossendorf e.V., Institute of Ion Beam Physics and Materials Research, 01328 Dresden, Germany

## Abstract

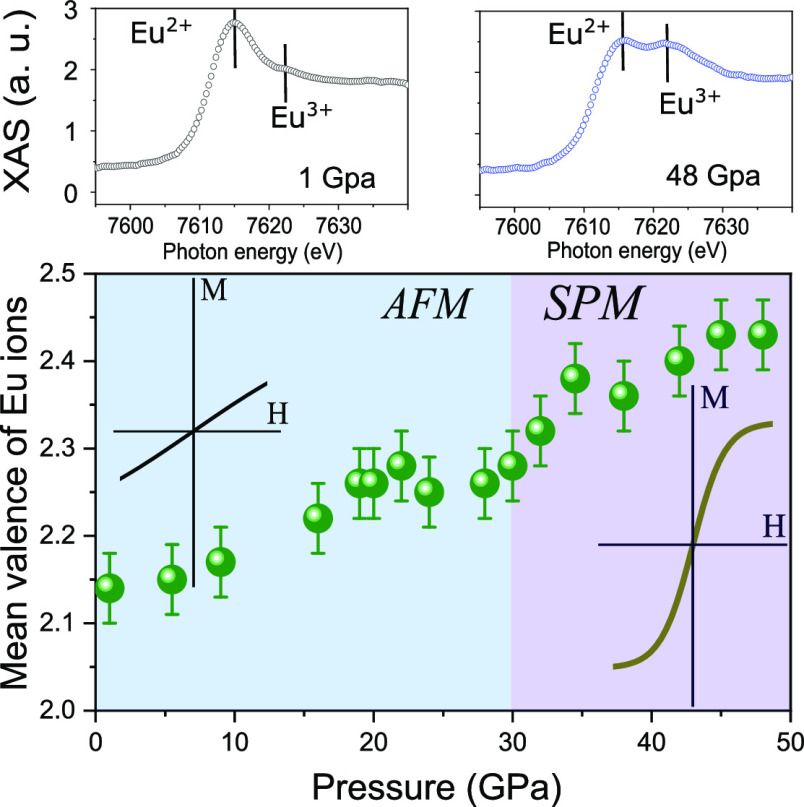

In rare-earth compounds with valence fluctuation, the proximity of the 4f level to the Fermi energy leads to instabilities of the charge configuration and the magnetic moment. Here, we provide direct experimental evidence for an induced magnetic polarization of the Eu^3+^ atomic shell with *J* = 0, due to intra-atomic exchange and spin–orbital coupling interactions with the Eu^2+^ atomic shell. By applying external pressure, a transition from antiferromagnetic to a fluctuating behavior in EuNiGe_3_ single crystals is probed. Magnetic polarization is observed for both valence states of Eu^2+^ and Eu^3+^ across the entire pressure range. The anomalous magnetism is discussed in terms of a homogeneous intermediate valence state where frustrated Dzyaloshinskii–Moriya couplings are enhanced by the onset of spin–orbital interaction and engender a chiral spin-liquid-like precursor.

Solid-state systems can undergo electronic transitions leading to intermediate or mixed valencies creating systems of ions with coexisting and, thus, correlated electronic configurations.^1^ Looking beyond the single ion, the understanding of collective phenomena, like magnetic ordering or superconductivity, in such strongly correlated electronic systems remains a major problem in condensed matter physics.^2^ If the two coexisting limiting configurations own qualitatively different magnetic states, a long-range ordered magnetic ground state may disappear or be replaced by a hidden or exotic magnetic order. Europium compounds with valence-fluctuating states provide a fruitful realization of such a valence transition. The divalent Eu^2+^ state with 4f^7^ (*L* = 0, *S* = 7/2, and *J* = 7/2) has a large pure spin-moment, while the trivalent Eu^3+^ with 4f^6^ configuration (*L* = 3, *S* = 3, and *J* = 0) is magnetically invisible. As the energy difference between Eu^2+^ and Eu^3+^ valence is not large,^3^ orbital intermixing can be achieved by applying external pressure or chemical substitution.^4−6^ Thus, coexistence of electronic configurations with energy differences in the thermal range can be achieved. By increase of the trivalent Eu at the expense of the divalent Eu, transitions from magnetically ordered to the paramagnetic state are expected, like in Ce and Yb-based materials.^7^ However, in the transition region the intermediate valency of the magnetic sites and a complex character of the intersite couplings may create novel magnetic behavior, as both are based on a strongly correlated electronic structure.^4,8,9^

The 4f^6^ configuration owns a spin-polarization with an identical but oppositely aligned orbital moment, and in addition, the *J* > 0 excitation of the 4f^6^-shell gives rise to Van Vleck (para)-magnetism.^10^ For the collective behavior, it has been theoretically suggested that such Van Vleck ions can contribute with a particular (anisotropic) intersite magnetic exchange,^11,12^ which could drive a hidden spin-ordering.^13^ Up to now, observation of hidden magnetic correlations between individual ions with 4f^6^ or also 5f^6^ configurations is rare^14−16^ and considered to originate from intersite exchange coupling mechanisms and the presence of a spin-polarized matrix acting on the Van Vleck ions.^14−18^ The admixture of the trivalent Eu also implies a modified orbital structure which could affect the magnetic ordering by atomic exchange and spin–orbital interactions. In this work, we investigate a magnetic europium compound with noncentrosymmetric lattice structure, which allows for the presence of the antisymmetric Dyzaloshinskii–Moriya interactions (DMIs).^19,20^ Magnetically, the EuNiGe_3_ exhibits a complex magnetic behavior below the Neel temperature (measured to be 13.2 K, see the Supporting Information), including an incommensurate helicoidal magnetic structure at 3.6 K.^31^ These couplings cause effective chiral couplings^21^ that frustrate homogeneous magnetic states and preclude conventional ordering according to the fundamental Landau theory of phase transitions.^22^ Instead, an intermediate chiral liquid-like or partially ordered state may appear,^23,24^ as experimentally found in chiral helimagnets like MnSi and FeGe under pressure.^25,26^

We report on a pressure-induced electronic phase transition in an antiferromagnetic and metallic compound, EuNiGe_3_,^27−31^ where a change of valence from dominating Eu^2+^ to an intermediate valence close to Eu^2.5+^ causes the appearance of a fluctuating magnetic state. This state is anomalous as it displays no magnetically homogeneous long-range order (LRO), but it is not paramagnetic either. Its thermal fluctuations can be characterized and quantified with the model for superparamagnetic (SPM) behavior. We argue that the observation of this unusual magnetism is evidence for a strongly correlated electronic system with partial magnetic order under the influence of chiral magnetic coupling caused by spin–orbit interactions. The tools used to detect the valence transition and the evolution of the fluctuating state are temperature- and pressure-dependent X-ray absorption spectroscopy (XAS) and X-ray magnetic circular dichroism (XMCD) at the Eu L_2_-edge, which are able to distinguish the polarization of the 5d orbital channels. Complementary to the Van Vleck paramagnetism characteristic for materials containing mainly Eu^3+^ ions, the polarization of 5d channels of Eu^3+^ states mirrors the magnetic behavior of Eu^2+^ under pressure, showing the same transition from AFM to an SPM-like behavior at about 30 GPa. In addition we observe a clear electronic phase transition of the Eu^3+^ as evidenced by a sudden linewidth change at the critical pressure. Our results provide direct evidence of intra-atomic exchange and spin–orbital interactions between the 5d channels of Eu^2+^ and Eu^3+^ contributions, which are essential to be considered when interpreting the physical properties of strongly correlated electronic systems.

The XAS spectra at the Eu L_2_-edge were taken at *T* = 8 K for a pressure range up to 48 GPa, as shown in Figure 1a. The quadrupolar (2p-4f) contributions which generally appear at the pre-edge^32^ were not observed, suggesting that the spectra are dominated by the dipolar contributions (2p_1/2_-5d_3/2_). The two contributions, shifted by ∼7.7 eV against each other (dashed lines in Figure 1a), belong to the 5d orbital channels of Eu^2+^ (4f^7^) and Eu^3+^ (4f^6^) states, respectively. This result, showing the coexistence of Eu^2+^ and Eu^3+^ levels, indicates that the valence fluctuation in EuNiGe_3_ takes place. A decrease of the Eu^2+^ content with a concomitant increase of the Eu^3+^ contribution is observed, evidencing the valence increasing under pressure. This valence change can be anticipated to increase as a function of pressure since electrons will be transferred out of the 4f shells into the conduction band.^33^

**Figure 1:**
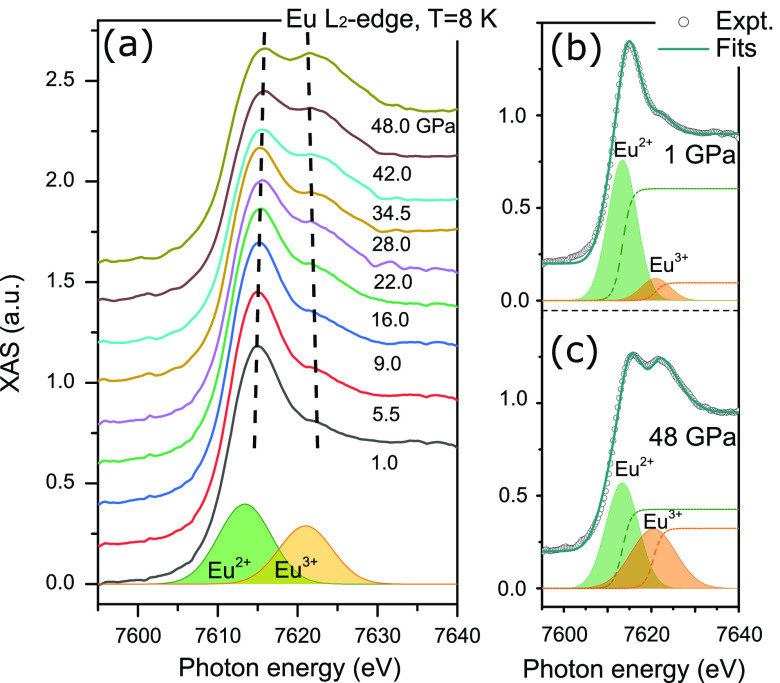
(a) The XAS of Eu L_2_-edge at 8 K under pressure up to 48 GPa, and the spectra of *P* = 1 GPa (b) and 48 GPa (c) fitted with the combination of the spectra of Eu^2+^ and Eu^3+^ with Gaussian-type lineshapes (thin solid lines). The dashed curves represent the integral background.

To evaluate the mean values of Eu valence, the spectra are analyzed by assigning Gaussian lineshapes to the Eu^2+^ and Eu^3+^ contributions, each with a *tanh*-type background, as shown in Figure 1b,c for the pressure of 1 and 48 GPa, respectively. The weighted sum, 86% Eu^2+^ and 14% Eu^3+^ for 1 GPa and 57% Eu^2+^ and 43% Eu^3+^ for 48 GPa, of the simulated curves describes the EuNiGe_3_ spectrum very well. The Eu mean valence can be derived from ν = 2 + *I*(Eu^3+^)/[*I*(Eu^3+^) + *I*(Eu^2+^)], where *I*(Eu^3+^) and *I*(Eu^2+^) denote the integrated intensities of the Eu^3+^ and Eu^2+^ components. Applying the fitting procedure, the Eu mean valence ν as a function of pressure have been extracted and are shown in Figure 3a. The results suggest that the Eu ion has a lower mean valence of ν = 2.13(3) at *T* = 8 K and 1 GPa and a much higher value of ν = 2.43(3) when the pressure increases to 48 GPa. The value at low pressure is in good agreement with the literature report ν = 2.09.^34^ A substantial enhancement of ν can be seen up to 48 GPa, except for *P* < 10 GPa below which a nearly constant value of ∼2.13(3) is preserved. This is in agreement with previous results showing no valence change up to 8 GPa according to the electrical resistivity measurements of EuNiGe_3_.^9^

The L_2_-edge XMCD spectra from the dipolar transition (2p^6^5d^0^ → 2p^5^5d^1^) reflect the polarization of the 5d empty-state orbitals in the conduction band. The pressure-dependent Eu L_2_-edge XMCD spectra and their line shape analysis (fwhm and energy position), which were recorded at *T* = 8 K and μ_0_*H* = 1.4 T and normalized to the XAS intensity, are presented in Figure 2a. Similar to the XAS, two well-defined peaks in the L_2_ transitions from Eu^2+^ and Eu^3+^ channels are clearly present in the XMCD spectra and are drastically affected by pressure. This undoubtedly indicates that the Eu 5d orbital are magnetically polarized in both Eu^2+^ and Eu^3+^ channels. The magnetic contribution from both channels can be well separated as shown in Figure 2b,c for pressure of 1 and 48 GPa, respectively. The area of the two peaks from Eu^2+^ and Eu^3+^ electronic states are denoted as A_2+_ and A_3+_, respectively, to further investigate the pressure dependence of the magnetic polarization from different 5d orbital channels. The peak positions of the XMCD spectra are slightly below the XANES peaks, similar to other Eu- and Sm-based fluctuating-valence materials of EuN,^15^ EuNi_2_P_2_,^35^ Sm_1–*x*_Gd_*x*_Al_2_,^36^ and SmB_6_.^37^ Moreover, through line shape analysis of the Eu^2+^ and Eu^3+^ resonances we observe that their resonant energy positions exhibit a linear dependence as a function of pressure, as shown in Figure 2d. They show a pressure-induced compression effect as the their energy difference diminishes from ∼9.9 eV at the lowest pressure of 1 GPa to ∼8.3 eV at the highest pressure of 48 GPa. During this compression, the full width at half-maximum (fwhm) reveals the occurrence of an electronic phase transition. While the fwhm of Eu^2+^ resonance remains unchanged for the whole pressure range, the fwhm of Eu^3+^ resonance exhibits a sudden increase at 30 GPa, from about 3 eV to about 6 eV. This electronic phase transition leads naturally to a strong enhancement of spin–orbital interactions due to the activation of a large orbital momentum characteristic of the Eu^3+^ electronic state.

**Figure 2:**
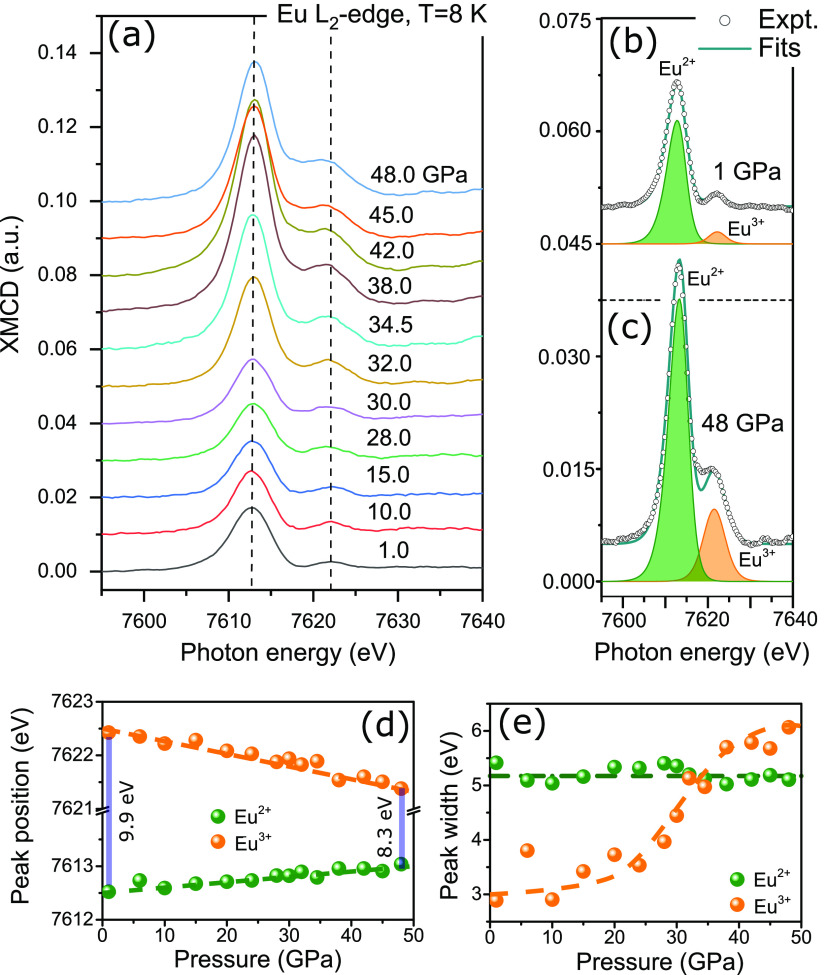
(a) Pressure-dependent Eu L_2_-edge XMCD spectra of bulk EuNiGe_3_ up to 48 GPa at *T* = 8 K and μ_0_*H* = 1.4 T, normalized to the XAS intensity, and the XMCD spectra at *P* = 1 (b) and 48 GPa (c) fits with the combination of the spectra of Eu^2+^ and Eu^3+^ with asymmetric double sigmoidal-type lineshapes. (d) Resonances peak position as a function of pressure, showing a pressure-induced compression effect. (e) The fwhm for the Eu^2+^ and Eu^3+^. At the critical pressure the fwhm of Eu^3+^ resonance exhibits a significant change, demonstrating the occurrence of a pressure-induced electronic phase transition.

The normalized XMCD intensity of *A* = *A*_2+_ + *A*_3+_ (Figure 3b) shows a completely different behavior when compared to the mean valence value. It remains unchanged up to 10 GPa (region I) and is slightly decreasing (10%) from 10 to 30 GPa (region II), followed by a sharp enhancement from 30 to 40 GPa (region III) with a factor of 3, and it finally dropped from 42 to 48 GPa (region IV). The slightly reduced magnetization in region II indicates the continuous increase of the Néel temperature for moderate pressure after 8 GPa.^9^ The jump of the macroscopic magnetization observed in region III clearly demonstrates the transition from AFM to a new magnetic phase at ∼30 GPa with a mean valence value of ν = 2.30. Following the increase of ν above 10 GPa, the magnetic contribution from Eu^3+^ increases from 0.10 at ∼10 GPa to 0.20 at ∼48 GPa, as shown in Figure 3c. This demonstrates that a stronger cumulative spin and orbital magnetic contribution from the Eu^3+^ state correlates with a higher Eu^3+^ occupation in EuNiGe_3_ under pressure.

**Figure 3:**
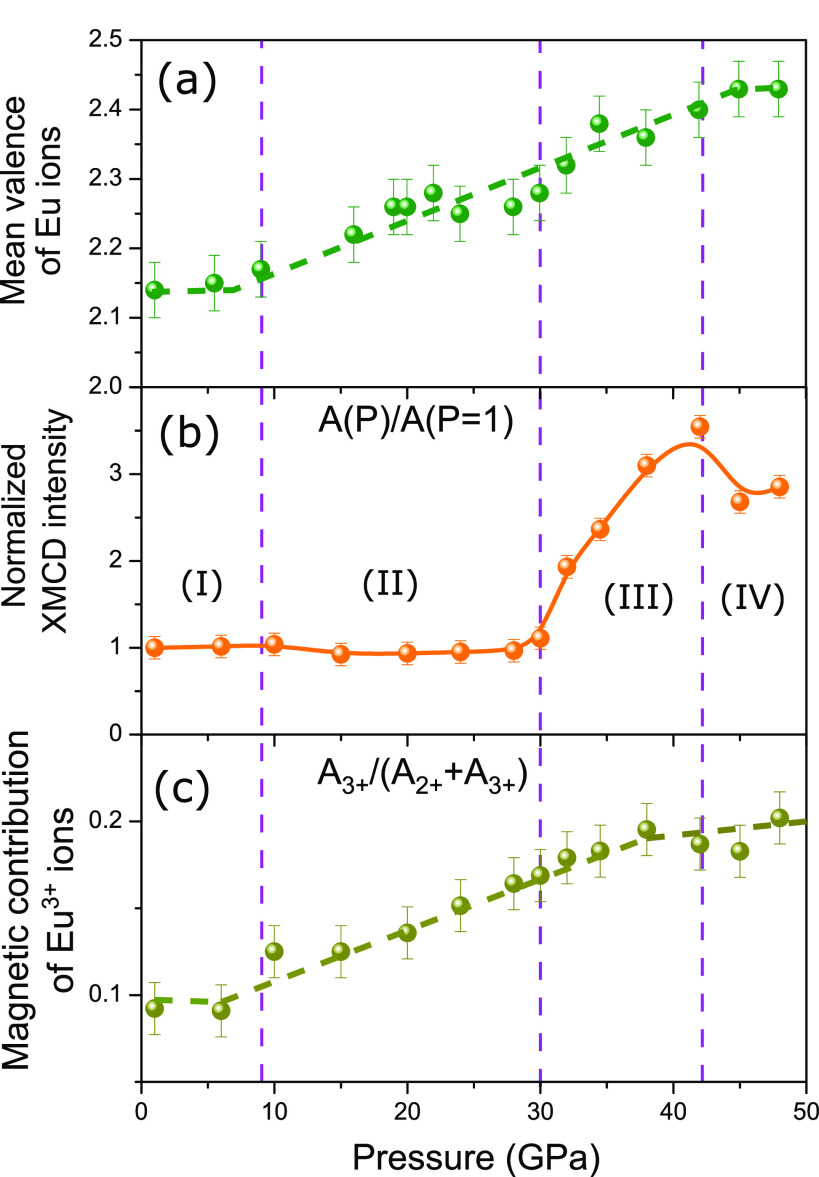
Eu mean valence ν (a), the normalized XMCD intensity (b) with a nonuniform behavior, and the relative magnetic contribution from 5d channel of Eu^3+^, *A*_3+_/(*A*_2+_ + *A*_3+_) (c), obtained as a function of the pressure at μ_0_*H* = 1.4 T and *T* = 8 K up to 48 GPa.

The magnetic contributions from Ni sites are negligible as probed by in situ high-pressure XAS and XMCD measured at the Ni K-edge up to 45.5 GPa, shown in Figures S6 and Figure S7 in the Supporting Information. Besides, there is no structural phase transition observed up to 57 GPa, as demonstrated by the in situ high-pressure X-ray diffraction results shown in Figure S8.

In addition to the large enhancement of the Eu magnetic polarization under pressure *P* > 30.0 GPa, an onset of a specific change of magnetic behavior is observed according to the field dependence of the XMCD intensity of Eu^2+^ at *P* = 32.0 and 34.5 GPa, as shown in Figure 4a. The profile of the XMCD spectra does not change with the field, suggesting the same magnetic behavior of the 5d channels from Eu^2+^ and Eu^3+^. The saturation tendency and the S-shape magnetic hysteresis loop indicates the onset of a thermally activated dynamics of the magnetic state above 30 GPa. By contrast, an almost linear curve is observed for *P* = 15.0 GPa in the AFM state, which is the ground state of the EuNiGe_3_ at ambient pressure.^9^ For simplicity, we analyze this anomalous behavior in terms of an SPM model by fitting the field-dependent XMCD with a Brillouin function.^38^ Note that the SPM model is most popular for the analysis of nanoparticles, whose magnetization can randomly flip direction within their characteristic relaxation times. However, short-range correlated spins may exhibit similar characteristic dynamics in magnetic systems. In particular, dense spin- or magnetic cluster-glasses, spin-density-wave order under random exchange or random-field, or other glassy magnetic systems do show such a behavior.

**Figure 4:**
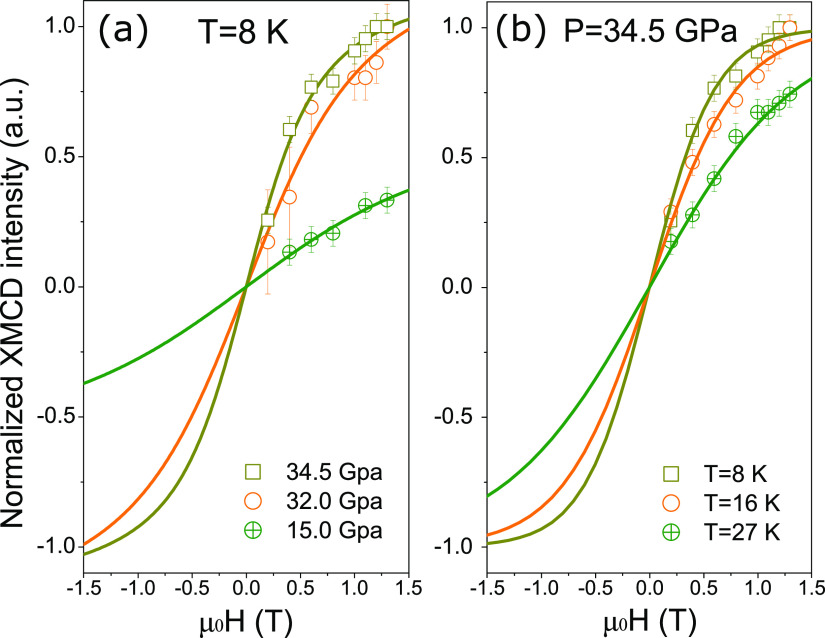
Field-dependent XMCD intensity of Eu, obtained at *T* = 8 K under pressure of 15.0, 32.0, and 34.5 GPa (a), and at *T* = 8, 16, and 27 K under pressure of 34.5 GPa (b). The lines are superparamagnetic fittings with Brillouin function.

The first scenario includes ferromagnetic correlation that is available in the magnetic ground state. In our case, the ground state is antiferromagnetic; therefore, dipolar interactions and an eventual percolation threshold cannot be supported. For the second scenario, a transition from spin density waves to a glassy behavior would require a breaking symmetry mechanism which involves impurities and/or random exchange fields. This can also be excluded, because the EuNiGe_3_ is a single crystal (no impurities) and the ground state is antiferromagnetic (no random fields). Similar arguments applied also for the third scenario. Instead, as we mentioned above, the EuNiGe_3_ crystal exhibits a noncentrosymmetric lattice structure, which allows for the presence of the antisymmetric DMIs.^19,20^ Then it is reasonable that a pressure-driven transition that involves valence fluctuations (Eu^2+^/Eu^3+^) under the presence of symmetry-breaking interactions causes a transition from AFM to an unconventional superparamgnetic state. This reflects short-range interaction of spins that are characterized by an effective magnetic moment which fluctuates with a paramagnetic long-range character.

Considering that the fluctuations are described by an effective moment, it defines the curvature of the field-dependent magnetization as a parameter. For 32.0 GPa, an equivalent of 4 magnetic Eu^2+^ states reproduce the data, whereas at 34.5 GPa an average number of 6.5 elemental moments result from the fitting to the data. These numbers, which reflect the ordered spins, are significantly higher as compared to a simple paramagnetic behavior where one magnetic atom would define the magnetization character. Corroborated also by the enhanced magnetization from 30.0 to 42.0 GPa (Figure 3c) one can suggest that the short-range magnetic interactions are strengthened by the lattice contraction, similar to that observed in EuX (X = Te, Se, S, O) monochalcogenides^8^ and Eu_0.5_Yb_0.5_Ga_4_.^4^ For a consistency check of the SPM behavior at high pressure, we plot in Figure 4b the XMCD dependence as a function of field for three different temperatures measured at 34.5 GPa. These curves also show the effect of enhanced thermally fluctuating moments by the change of curvature with the typical thermally activated SPM-like dynamics.

The paramagnetic behavior of EuNiGe_3_ at ambient pressure and above the Néel temperature is confirmed according to the temperature-dependent magnetic moments as shown in Figure 5a. For each temperature the XMCD has been measured at Eu M_4,5_-edges in an external field of μ_0_*H* = ± 8 T applied along the *c*-axis of the crystal. The magnetic moments have been retrieved through the sum rules^39^ analysis applied to the XMCD spectra (not shown). The line in Figure 5a represents a plot of the Brillouin function for parameters characteristic to a divalent Eu. The agreement between the model and the measured magnetic moment as a function of temperature confirms the paramagnetic behavior of the magnetization above the ordering temperature. Below the Néel temperature, the hysteresis loop at *T* = 8 K (inset of Figure 5a) confirms its AFM ground state at low temperatures. In Figure 5b we show the XMCD intensity (at the L_2_ edge) of Eu^2+^ as well as Eu^3+^ which were recorded under *P* = 48.0 GPa for an external field of μ_0_*H* = 1.4 T and for temperatures ranging from 8 to 250 K. The normalized values of A_2+,3+_(*T*)/A_2+,3+_(8 K) deviate significantly from the ideal paramagnetic behavior, confirming an SPM character. Also, the XMCD intensity of the Eu^3+^ follows closely the behavior of Eu^2+^, which suggests a strong intra-atomic exchange interaction in the valence-fluctuating EuNiGe_3_.

**Figure 5:**
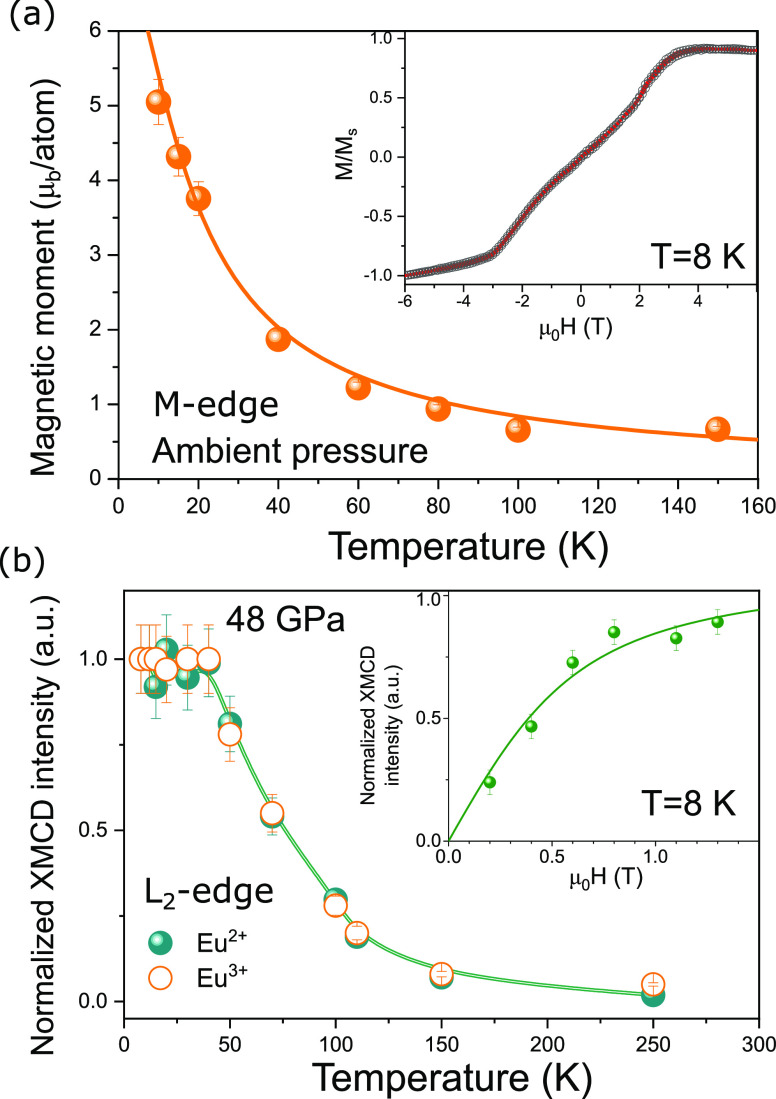
(a) Temperature dependence of the magnetic moment of Eu at ambient pressure. The line represents a plot of the Brillouin function for parameters characteristic to the divalent Eu. Inset: AFM-type hysteresis loop measured at 8 K. (b) Eu^2+^ and Eu^3+^ XMCD intensities (normalized to the value of 8K) measured at L_2_-edge (solid and open circles) obtained at *P* = 48.0 GPa from 8 to 250 K, and the line is a guide to the eyes. Inset: field-dependent XMCD intensity of Eu^2+^.

The mechanism of the SPM-like state correlates with the onset of the electronic phase transition which leads to the onset of the spin–orbit coupling trough populating the Eu^3+^ electronic state. EuNiGe_3_ has an acentric polar crystal structure (of BaNiSn_3_ structure-type: space group *I*4*mm*, No. 107) which causes the appearance of frustrated chiral Dzaloshinskii–Moriya interactions (DMIs)^21^ that are enhanced by the onset of the orbital moment of the Eu^3+^ at the transition pressure. This mechanism is present in EuNiGe_3_ by symmetry, and an unconventional transition from AFM to another magnetic LRO or the paramagnetic state is expected to display an intermediate or meso-phase with fluctuating larger magnetic units than the paramagnetic ions. The chiral DMIs are always present; thus, they are active also in the homogeneous intermediate valence state, where the on-site fluctuations between 4f^7^ and 4f^6^ configurations are so fast that magnetic properties are determined by the magnetic moments of a smeared state with fractional valence on site and its intersite exchange.

To conclude, element and orbital selective XAS and XMCD measurements on Eu L_2_ absorption edges under pressures up to 48.0 GPa show a prominent valence change in EuNiGe_3_ from Eu^2+^ toward Eu^3+^ as a function of pressure. Both the 5d channels of the Eu^2+^ and Eu^3+^ contributions are magnetically polarized, and an electronic phase transition is observed at 30 GPa as a sudden increase of the resonance line width of the Eu^3+^. Concomitantly, a magnetic transition to an anomalous state of slow and large thermal fluctuating moments is observed. The chiral magnetic exchange and a precursor state is identified as the underlying mechanism for this anomalous state. In EuNiGe_3_, the 5d orbital channels of Eu^3+^ has *J* = 0 ground state and therefore is not responsive to the applied magnetic field. The polarization of 5d orbital channels of Eu^3+^, which is intimately bound to that of Eu^2+^ for all temperature and pressure ranges, suggests intra-atomic exchange interactions to the Eu^2+^ in valence-fluctuating EuNiGe_3_. Such strong intra-atomic exchange and spin–orbit interactions need to be considered for future theoretical investigations of Eu- and other rare earth-based materials with a valence fluctuating state.

## Methods

Single crystals of EuNiGe_3_ were grown by using a high-temperature solution growth method with In as a solvent, as described in more details in refs (30, 40, and 41). The XAS and XMCD spectra at the Eu L_2_-edge and Ni K-edge have been measured at the ODE beamline^42^ at synchrotron-SOLEIL, France to probe the pressure-dependent local electronic configuration and 5d magnetism of Eu ions. Micrometer-sized powders ground from a high-quality single-crystal EuNiGe_3_, together with the pressure-transmitting medium silicon oil, was pressurized up to 48 GPa in a diamond-anvil cell. The pressure was measured using a ruby fluorescence scale. XMCD spectra were obtained through the difference of XAS spectra measured under the magnetic field up to μ_0_*H* = 1.4 T, applied parallel or antiparallel to the beam helicity. The XMCD at the Eu M-edges were measured at the VEKMAG end-station^43^ installed at the PM2 beamline of the synchrotron facility BESSY II, under external magnetic fields up to μ_0_*H* = 8 T applied along the *c*-axis of the single crystal. The in situ high-pressure X-ray diffraction measurement was performed with an angle-dispersive synchrotron X-ray diffraction mode (AD-XRD) at the BL04 beamline of the ALBA.

## Supporting Information Available

Details of the preparation of EuNiGe_3_ single crystal under investigation and its anisotropic magnetic properties in ambient pressure conditions; element-specific XMCD measurements for Eu and Ni, utilizing soft X-ray spectroscopy at the ambient pressure; high-pressure XMCD at Ni K-edge; and high-pressure X-ray diffraction results (PDF)
