# Supplementary material for: Evidence of the Anomalous Fluctuating Magnetic State by Pressure-Driven 4f Valence Change in EuNiGe3
Source: J Phys Chem Lett. 2023 Jan 24;14(4):1000–6. doi: 10.1021/acs.jpclett.2c03569 (PMC9900636; doi:10.1021/acs.jpclett.2c03569)
Supplement: Supplementary file 1 — jz2c03569_si_001.pdf [file jz2c03569_si_001.pdf]

Supplementary Information

Evidence of The Anomalous Fluctuating Magnetic State by Pressure Driven 4f Valence Change in $EuNiGe_3$

K.Chen[1,*], C.Luo[2], Y.Zhao[3] F.Baudelet[4], A.Maurya[5], A.Thamizhavel[5], U.K.Rößler[6], D.Makarov[7], F.Radu[2,*]

1 National Synchrotron Radiation Laboratory, University of Science and Technology of China, Hefei 230026, Anhui, China
2 Helmholtz-Zentrum Berlin für Materialien und Energie, Albert-Einstein-Strasse 15, 12489, Berlin, Germany
3 Center for High Pressure Science and Technology Advanced Research (HPSTAR), Shanghai 201203, China
4 Synchrotron SOLEIL, L'Orme des Merisiers, Saint-Aubin-BP48, 91192 GIF-sur-Yvette Cedex, France
5 Department of Condensed Matter Physics and Materials Science, Tata Institute of Fundamental Research, Colaba, Mumbai 400005, India
6 Leibniz-Institut für Festk\"{o}rper- und Werkstoffforschung Dresden e. V. (IFW Dresden), 01069 Dresden, Germany
7 Helmholtz-Zentrum Dresden-Rossendorf e.V., Institute of Ion Beam Physics and Materials Research, 01328 Dresden, Germany

The details of the crystal growth and the anisotropic magnetic properties are discussed in Ref. [S1] of the main manuscript. Here we describe the preparation of $EuNiGe_3$ single crystal under investigation and its anisotropic magnetic properties in ambient pressure conditions. Also, we show for one temperature and at the ambient pressure the element specific XMCD measurements for Eu and Ni, utilizing soft x-ray spectroscopy. High-pressure X-ray diffraction results showing no phase transition up to 57.3 GPa.

Supplementary Note 1. Crystal growth and sample characterization by x-ray diffraction

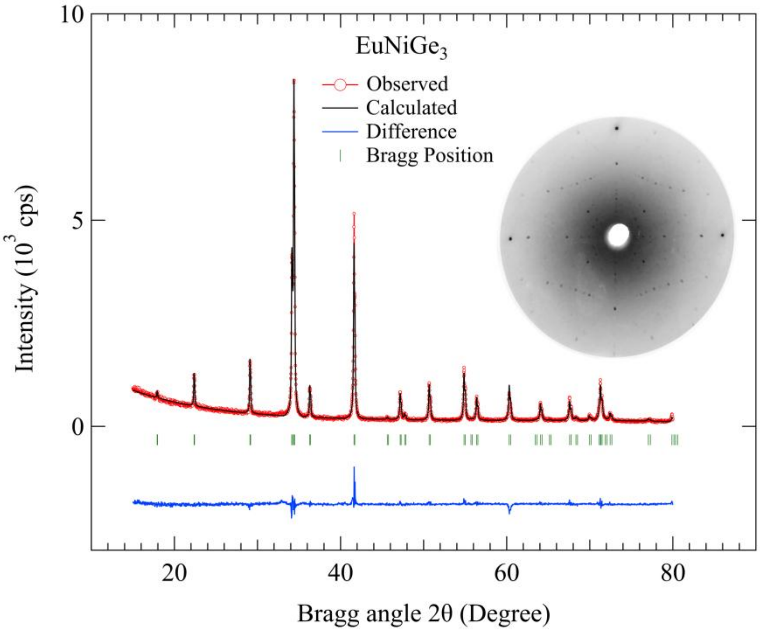

Figure S1: Room temperature powder x-ray diffraction pattern along with the Rietveld refinement. The inset shows the Laue diffraction pattern corresponding to (100) plane of the tetragonal I4mm space group. Well defined Laue diffraction pattern ascertains the good quality of the single crystal.

Single crystals of $EuNiGe_3$ were grown by high temperature solution growth using molten In as the flux. To start with, a polycrystalline sample of $EuNiGe_3$ was prepared by arc melting method and then this polycrystalline specimen along with excess indium (In) was placed in a recrystallized alumina crucible. The crucible was subsequently sealed in a quartz ampoule and heated to a maximum temperature of 1100 °C and held at this temperature for about 24 h, for proper homogenization. Then the furnace was cooled down to 600 °C at a rate of 2 °C/h where the excess indium was centrifuged. The quality of the crystals has been characterized by powder x-ray diffraction analysis, after powdering the some of the single crystals. The x-ray diffraction pattern was clean without any impurity peaks, confirming the space group *I4mm* and the lattice constant. Furthermore, the Laue diffraction was performed to orient the crystal along the crystallographic directions. Well defined Laue diffraction pattern confirms the good quality of the single crystal. The x-ray diffraction pattern, the Laue diffraction pattern, as well as the Rietveld refinement of the scattering pattern are shown in Fig. S1.

Supplementary Note 2. Magnetic characterization of the $EuNiGe_3$ at ambient pressure

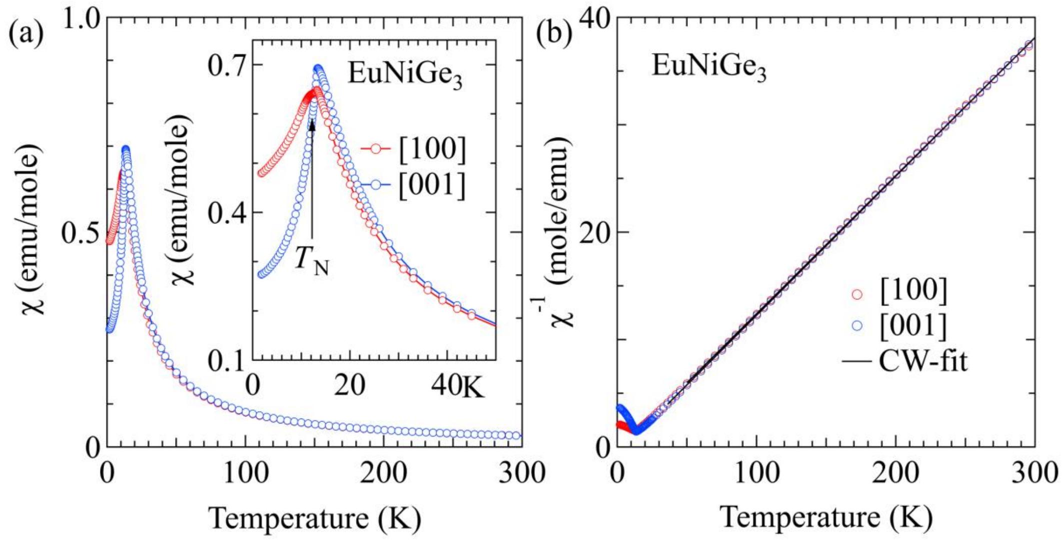

Figure S2: (a) Temperature dependence of magnetic susceptibility along the two principal crystallographic directions. The inset shows the low temperature part, the Neel temperature is indicated by an arrow. (b) Inverse magnetic susceptibility, the solid lines represent the Curie-Weiss fit.

The temperature dependent magnetic susceptibility from 2 to 300 K is shown in Fig. S2(a). It is evident from the figure that in the paramagnetic region, the susceptibility follows the Curie-Weiss behavior, and a sharp cusp is observed at T_N = 13.2 K. The magnetic susceptibility drops rapidly for H // [001] direction confirming the easy axis of magnetization. In the Fig. S2(b) we show the inverse magnetic susceptibility data. A Curie-Weiss fit from 50 K to 300 K yields the paramagnetic Weiss temperature and the effective magnetic moment

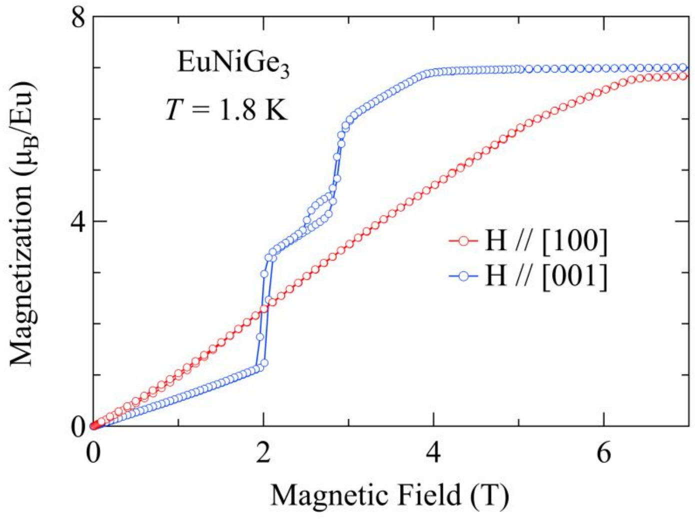

Figure S3: Isothermal magnetization measured at T = 2 K along the two principal crystallographic directions. The magnetization along H // [001] exhibits multiple metamagnetic transitions and saturates at around 4 T, while the magnetization along [100] direction is linear and saturates to 7 μ_B/Eu at around 6 T.

(μ_{eff}) as 3.4 K and 7.89 μ_B/Eu, respectively for H // [100] and 5.1 K and 7.90 μ_B/Eu for H // [001] direction. The effective moment value confirms that Eu exhibits a divalent state at ambient pressure conditions. The isothermal magnetization measured at T = 2 K along the two principal crystallographic directions, is shown in Fig. S3. For H // [100], the magnetization varies linearly with field up to 6.2 T, with a slight change of slope at around 4.6 T. For H // [001] the magnetization increases linearly with field and undergoes a spin-flop transition at 2 T, followed by another spin re-orientation at 3 T, and finally another spin re-orientation at 4.1 T where it saturates to 7 μ_B/Eu.

Supplementary Note 3. Eu and Ni L-edge XAS and XMCD spectra at ambient pressure and low temperature

Measurements of the single crystal have been performed by soft-x-ray absorption spectroscopy at the Eu M edges and Ni L edges. In order to access uncontaminated states of the probing elements, the sample was cleaved in-situ. This has been accomplished by fixing a 2mm in diameter Cu post on the crystal surface which has been detached in UHV conditions using a hard metal hit. The temperature of the sample was set to ~12.5 K and the x-ray absorption spectra (XAS) were collected in external magnetic fields oriented perpendicular to

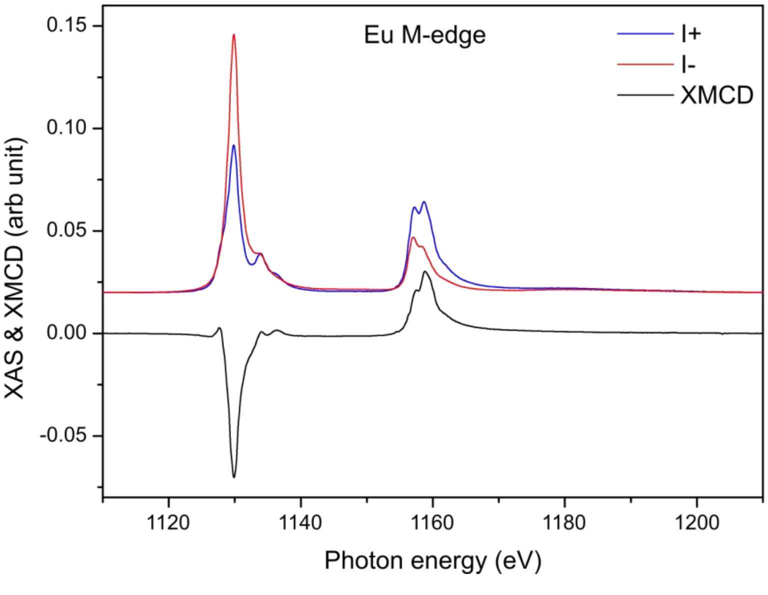

Figure S4: XAS and XMCD spectra of Eu M-edge, measured at 10K, 8T for EuNiGe3.

the sample surface, along the c-axis.

In Fig. S4 we show the XAS spectra measured in Total electron yield mode for a magnetic field of μ_0H=8T (blue line) and for an opposite magnetic field of μ_0H=-8T. We observe a large difference of the spectra which provides, through their subtraction, the x-ray magnetic circular dichroism (XMCD) signal, which is shown as black line in the same figure. The lineshape of both XAS and XMCD are characteristic of a divalent Eu.

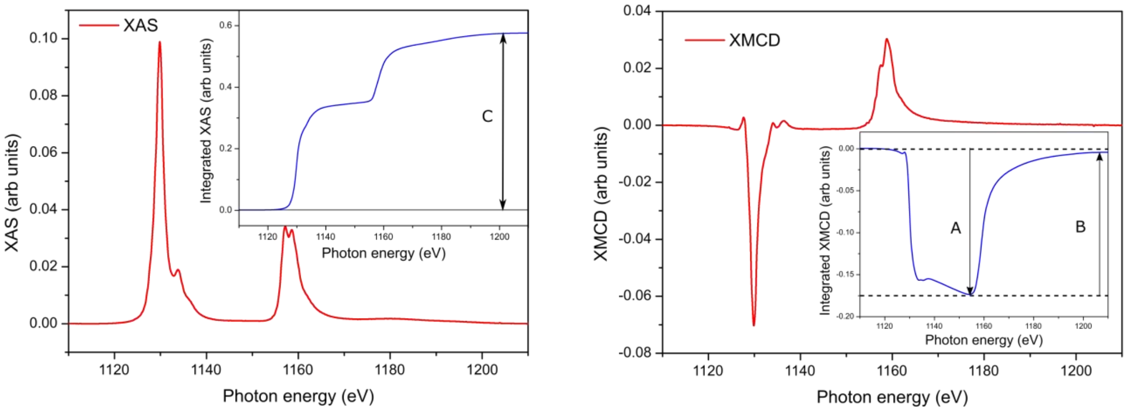

Figure S5: Sum rule analysis of Eu M-edge, (left) XAS spectra and the integrated XAS spectra and (right) XMCD and the integrated XMCD spectra.

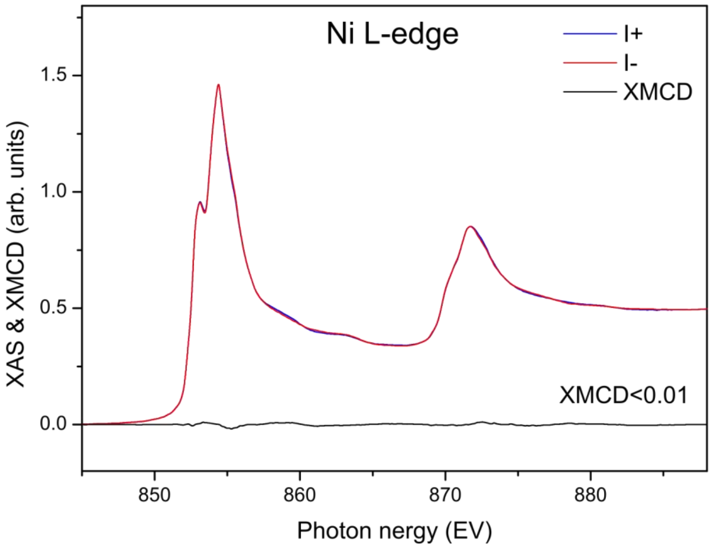

Figure S6: XAS and XMCD spectra of Ni L-edge, measure at 10 K, 8T for $EuNiGe_3$. The very small XMCD intensity (<1%) suggests for the non-magnetic Ni sites in $EuNiGe_3$.

By applying the sum rules (see Fig. S5) as described in Ref.[S3-S4] it is possible to determine the effective spin magnetic moment m^{eff}_S and the orbital magnetic moment m_L :

$$M^{eff}_{S} = 2 < S_Z > +7 < T_Z > = -\frac{2A-3B}{2C} \bullet n_{4f}$$

$$M_L = -\frac{A+B}{C} \bullet n_{4f}$$

where A and B are the integrated XMCD intensity at the Eu M_5 and M_4 edges, respectively (Fig. S5 right panel). n_{4f} is the number of holes in the 4f shell of Eu, C is the sum of the integrated XAS intensity at Eu M_5 and Eu M_4 edges (Fig. S5 left panel), T_Z is the magnetic-

dipole operator of Eu. Assuming T_Z=0 for Eu2+ and considering n_{4f}=7 we obtained an almost zero orbital moment (M_L ~ 0.01 μB) and M_S=5.2(0.3) μB per Eu^{2+} ion at 12.5 K.

Ni L-edge XAS and XMCD spectra were recorded at 12.5 K, for magnetic fields of μ_0H=+/-8T, applied along the c-axis (Fig. S6). The XAS curves are very similar to each other, suggesting for non-magnetic Ni atoms in EuNiGe3, as proposed in Ref. [S2]. The XMCD signal, which is less than 1% of the XAS intensity, is negligible as compared to the bulk Ni. The quench of the magnetization of Ni atoms confirms that the magnetic properties are ruled by the Eu atoms, alone in $EuNiGe_3$.

Supplementary Note 4. Ni K-edge XAS and XMCD spectra at high pressure and low temperature

The in-situ high pressure XAS data of Ni K-edge up to 45.5 GPa and the selected XMCD spectra (See Figure S7), which was performed at ODE beamline (H=1.3T and 10 K) at synchrotron Soleil, showing no magnetic contribution from Ni sites. Up on pressure increasing, the profile of the Ni K-edge XAS kept unchanged suggests for no structure transition of EuNiGe3 materials.

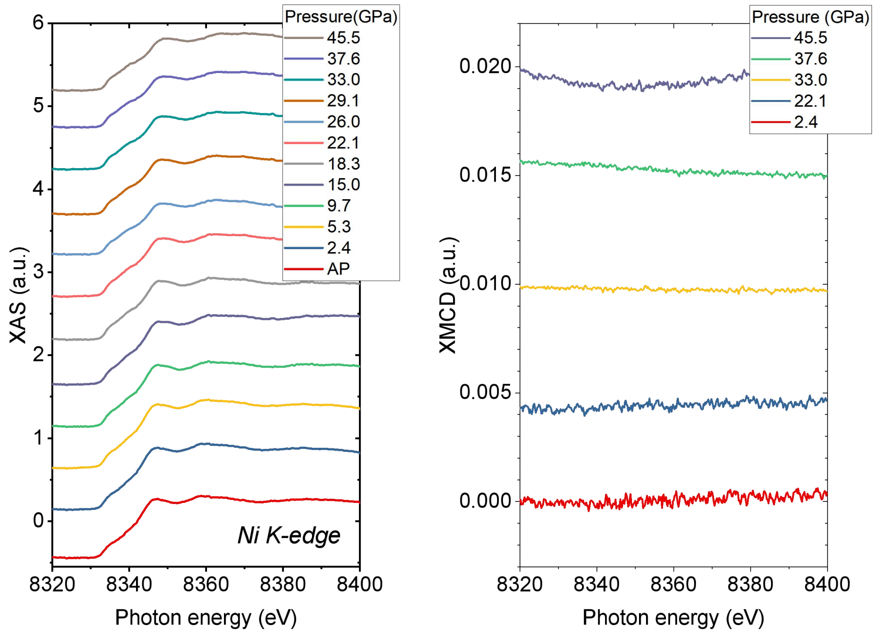

Figure S7: In-situ high pressure XAS data of Ni K-edge up to 45.5 GPa (a) and the selected XMCD spectra(b) showing no magnetic contribution from Ni sites.

Supplementary Note 5. High-Pressure X-ray Diffraction Results

The *in situ* high-pressure X-ray diffraction (λ = 0.4246 Å) measurement was performed with an angle-dispersive synchrotron X-ray diffraction mode (AD-XRD) at beamline BL04 beamline of the ALBA. The as-prepared samples were loaded into a gasketed diamond anvil cell (DAC) with silicon oil as a pressure-transmitting medium for the highest pressure to 57.3 GPa. *In-situ* high pressure XRD measurements are performed and selected patterns are

presented in **Supplementary Figure S8** (a), which did not reveal any crystallographic symmetry change up to 57.3 GPa. Nevertheless, one peak splitting near 12-degree occurred at 32.3 GPa, indicating a subtle structural deformation. The V-P plots of $EuNiGe_3$ was fitted with a third-order Birch-Murnaghan equation of state (EOS), as shown in **Supplementary Figure S8**(b), which yielded a bulk modulus of B_0 =79.0(8) GPa with B′= 8.8 up to the highest pressure. No phase transition is observed but accompanied with a lattice distortion above 32.3 GPa.

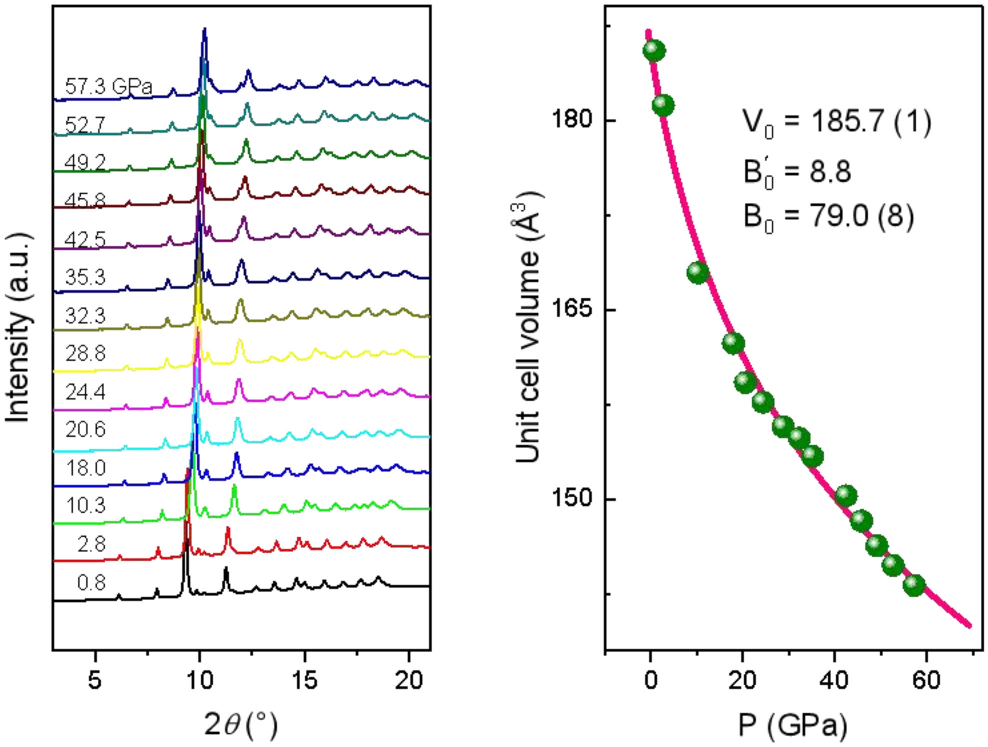

Figure S8: Selected patterns of in-situ high pressure XRD data up to 57.3 GPa (a) and the V-P plots of EuNiGe3 fitted with a third-order Birch-Murnaghan equation of state(b).

References:

[S1]: Maurya, A.; Bonville, P.; Thamizhavel, A.; and Dhar, S. K.; EuNiGe3, an anisotropic antiferromagnet, J. Phys.: Condens. Matter **2014**, *26*, 216001.

[S2]:Goetsch, R. J.; Anand, V. K.; and Johnston, D. C.; Antiferromagnetism in $EuNiGe_3$, Phys. Rev. B **2013**, *87*, 064406.

[S3] Thole, B. T.; Carra, P.; Sette, F.; and van der Laan, G.; X-ray circular dichroism as a probe of orbital magnetization, Phys. Rev. Lett. **1992**, *68*, 1943.

[S4] Carra, P.; Thole, B. T.; Altarelli, M.; and Wang, X.; X-ray circular dichroism and local magnetic fields, Phys. Rev. Lett. **1993**, *70*, 694.
